# *HvFT1* polymorphism and effect—survey of barley germplasm and expression analysis

**DOI:** 10.3389/fpls.2014.00251

**Published:** 2014-06-06

**Authors:** Jorge Loscos, Ernesto Igartua, Bruno Contreras-Moreira, M. Pilar Gracia, Ana M. Casas

**Affiliations:** ^1^Department of Genetics and Plant Production, Estación Experimental de Aula Dei, Consejo Superior de Investigaciones CientíficasZaragoza, Spain; ^2^Fundación ARAIDZaragoza, Spain

**Keywords:** barley, flowering time, copy number variation, *HvFT1*, *HvCEN*

## Abstract

Flowering time in plants is a tightly regulated process. In barley (*Hordeum vulgare* L.), *HvFT1*, ortholog of *FLOWERING LOCUS T*, is the main integrator of the photoperiod and vernalization signals leading to the transition from vegetative to reproductive state of the plant. This gene presents sequence polymorphisms affecting flowering time in the first intron and in the promoter. Recently, copy number variation (CNV) has been described for this gene. An allele with more than one copy was linked to higher gene expression, earlier flowering, and an overriding effect of the vernalization mechanism. This study aims at (1) surveying the distribution of *HvFT1* polymorphisms across barley germplasm and (2) assessing gene expression and phenotypic effects of *HvFT1* alleles. We analyzed *HvFT1* CNV in 109 winter, spring, and facultative barley lines. There was more than one copy of the gene (2–5) only in spring or facultative barleys without a functional vernalization *VrnH2* allele. CNV was investigated in several regions inside and around *HvFT1*. Two models of the gene were found: one with the same number of promoters and transcribed regions, and another with one promoter and variable number of transcribed regions. This last model was found in Nordic barleys only. Analysis of *HvFT1* expression showed that association between known polymorphisms at the *HvFT1* locus and the expression of the gene was highly dependent on the genetic background. Under long day conditions the earliest flowering lines carried a sensitive *PpdH1* allele. Among spring cultivars with different number of copies, no clear relation was found between CNV, gene expression and flowering time. This was confirmed in a set of doubled haploid lines of a population segregating for *HvFT1* CNV. Earlier flowering in the presence of several copies of *HvFT1* was only seen in cultivar Tammi, which carries one promoter, suggesting a relation of gene structure with its regulation. *HvCEN* also affected to a large extent flowering time.

## Introduction

In temperate cereals, like barley (*Hordeum vulgare* L.) and wheat (*Triticum aestivum* L.), flowering is regulated by the integration of two seasonal signals (Laurie, 2009): photoperiod (day length) and vernalization (prolonged exposures to low temperatures). Flowering time is also closely linked with agronomic performance. Plants must flower at the appropriate time of the year, when conditions are most favorable for pollination, seed development and high grain yield.

The responses to day length and temperature serve to classify barley varieties according to their adaptation pattern. Based on the response to day length, varieties are divided into photoperiod-sensitive (long days accelerate flowering) or -insensitive (plants flower almost independently of the day length). Based on the response to vernalization, barley varieties are classified as winter (vernalization is required for timely flowering) or spring (flowering irrespective of vernalization), although the presence of an allelic series at *VrnH1* produces intermediate genotypes (Hemming et al., 2009). Usually, winter varieties are sown in autumn, spring varieties in winter and spring, and there is a third category known as facultative varieties, that can be sown anytime. Several major genes are the main responsible for the responses to photoperiod and vernalization, which are described next.

Allelic differences in the photoperiod genes *PpdH1* and *PpdH2* are associated with natural variation in the response to day length. *PpdH1* (a member of the *Pseudo Response Regulator* family) is part of the circadian clock of the plant and promotes flowering under long days (Turner et al., 2005). Recessive mutations in the *PpdH1* gene result in delayed flowering under long days (Turner et al., 2005; Hemming et al., 2008). The *PpdH1* gene acts in parallel to *HvCO1* (Campoli et al., 2012), which is one of the barley homologs of the *Arabidopsis* (*Arabidopsis thaliana*) *CONSTANS* (*CO*) gene. Overexpression of *HvCO1* results in the up-regulation of *HvFT1* (the ortholog in barley of the *Arabidopsis FLOWERING LOCUS T*, or *FT*) and the acceleration of flowering (Campoli et al., 2012). *PpdH2* has been long acknowledged as the responsible for acceleration of flowering in response to short photoperiod, although its role is being re-defined (Casao et al., 2011). It is a paralog of *HvFT1*, (alternatively named *HvFT3* by Faure et al., 2007 and Kikuchi et al., 2009). Its effect on flowering is not as strong as *HvFT1* and it seems to be restricted to winter genotypes under short days or long days without vernalization (Casao et al., 2011). Another paralog of *HvFT1* with a large effect on time to flowering, particularly at Mediterranean latitudes (Boyd et al., 2003; Cuesta-Marcos et al., 2008), is *HvCEN*, and its two main haplotypes are differentially distributed over spring and winter barley varieties (Comadran et al., 2012).

Natural variation in barley vernalization requirement is mainly found in the vernalization loci *VrnH1* (Trevaskis et al., 2003; Yan et al., 2003), *VrnH2* (Yan et al., 2004), and *VrnH3* (Yan et al., 2006). The *VrnH1* gene is closely related to the *Arabidopsis* gene *APETALA1* (*AP1*), responsible for the transition from the vegetative to the reproductive stage (Trevaskis et al., 2007). Different alleles of *VrnH1* have been identified, with insertions or deletions in the first intron of the gene (von Zitzewitz et al., 2005; Cockram et al., 2007; Hemming et al., 2009), affecting the length of the optimum vernalization period. Alleles lacking large sections of the ~11 kb intron are more active and are associated with earlier flowering without vernalization, whereas alleles lacking small segments are associated with only a moderate increase in *VrnH1* activity and weaker promotion of flowering (Szűcs et al., 2007; Hemming et al., 2009). *VrnH2* includes three closely related genes designated as *HvZCCTa-c*, which are characterized by a putative zinc finger and a CCT-domain. *VrnH2* is considered to play the role of repressor of flowering and it has been shown that deletions of all the three *HvZCCT* genes result in spring growth habit (Karsai et al., 2005; Trevaskis et al., 2006). Finally, *VrnH3* was shown to correspond to *HvFT1*, the ortholog of the *Arabidopsis FT* gene (Yan et al., 2006; Faure et al., 2007).

*FT* is considered as the main flowering integrator of the photoperiod and vernalization pathways in both monocot and dicot species (Turck et al., 2008). In barley, the most accepted hypothesis for the regulation of *HvFT1* establishes that, during the fall, when temperate cereals germinate, *HvFT1* is repressed by *VrnH2* (Yan et al., 2006; Hemming et al., 2008; Distelfeld et al., 2009). During winter, vernalization up-regulates *VrnH1* (Trevaskis et al., 2006; Oliver et al., 2009), which results in the repression of *VrnH2* in the leaves and, consequently, the activation of *HvFT1* transcription in the spring (Loukoianov et al., 2005; Trevaskis et al., 2006; Hemming et al., 2008; Chen and Dubcovsky, 2012). The precise molecular mechanisms of action of these genes are still the object of numerous studies in barley and other cereals. *FT* transcription is induced in the leaves and it has been demonstrated in different species that the encoded protein travels through the phloem to the stem apical meristem, where it plays a central role in triggering flowering (Corbesier et al., 2007; Tamaki et al., 2007). In *Arabidopsis*, FT interacts with the bZIP transcription factor FD and up-regulates the expression of the meristem identity gene *AP1* at the shoot apex (Abe et al., 2005; Wigge et al., 2005). A similar interaction has been reported in wheat, where the orthologous FT protein interacts with an FD-like protein and has the ability to bind *in vitro* the promoter of *VrnH1*, the wheat homolog of *AP1* (Li and Dubcovsky, 2008).

Ample natural variation in the *HvFT1* gene has been found, with polymorphisms reported in the promoter and in the first intron. This variation has been linked to differences in flowering phenotypes in a number of studies (Yan et al., 2006; Hemming et al., 2008; Casas et al., 2011). It seems clear now that, in *Arabidopsis*, the *FT* promoter and first intron contain cis-regulatory sites that are important for its transcriptional regulation (Tiwari et al., 2010). However, the *FT1* regulatory regions of barley and wheat are not as well characterized. Yan et al. (2006) found an association between growth habit and mutations in the first intron, but the sequencing of the *HvFT1* alleles from populations previously used to map QTL for flowering time (Hemming et al., 2008) failed to reveal any significant association between the two linked SNPs in intron 1 and flowering time. Further results reported in other surveys of *HvFT1* allelic variation (Cuesta-Marcos et al., 2010; Casas et al., 2011) were also in disagreement with Yan et al. (2006) regarding the direction of the effect assigned to the functional polymorphism in the first intron. Yan et al. (2006) also identified two promoter haplotypes, characterized by seven linked SNPs and two indels in the first 550 bp upstream of the start codon. Using primers specific to differentiate those indels, Casas et al. (2011) analyzed natural variation for the promoter haplotypes (135–146 vs. 139–142 bp) and the intron 1 haplotypes (AG/TC) in a collection of barley landraces. In that study the intron AG haplotype was clearly associated with later flowering than the TC haplotype. The results for the promoter haplotypes hinted at a role of these polymorphisms on flowering time, but of lesser magnitude than intron polymorphism. The combination of the 135–146 promoter with the TC intron was associated with earliest flowering (Casas et al., 2011; Ponce-Molina et al., 2012). Further evidence from other populations (Nitcher et al., 2013) confirmed the description of promoter haplotypes as “early” (135–146) and “late” (139–142). Another SNP in the promoter of the *HvFT1* gene, upstream of the studied region was also suggested to have an additional role on flowering time (Cuesta-Marcos et al., 2010; Casas et al., 2011).

The scope of *HvFT1* polymorphism has been recently widened even further by including copy number variation (CNV), first described by Nitcher et al. (2013). Recently, this type of polymorphism has been proposed as a key contributor to intra-species genetic variation, along with SNPs and indel polymorphisms. Some data suggest that CNV mainly affects the members of large families of functionally redundant genes and that the effects of individual CNV events on phenotype are usually modest (Zmieńko et al., 2014). Nevertheless, there are many cases in which CNVs for specific genes have been linked to important traits such as flowering time and plant height and resistance (Zmieńko et al., 2014). Regarding *HvFT1*, it has been reported recently that a genotype with high gene copy number (BGS213, derived from cultivar Tammi) was responsible for early flowering and an epistatic override of winter growth habit, caused by the combination of *vrnH1* and *VrnH2* alleles (Nitcher et al., 2013). This study pursues to carry out a comprehensive survey of *HvFT1* polymorphisms, including CNV, in barley accessions of different origins and germplasm groups. Also, we aim to provide further new information on gene expression and phenotypic effects of contrasting genotypes at *HvFT1*.

## Materials and methods

### Plant materials

#### HvFT1 polymorphism

A set of 109 genotypes was used to survey the polymorphisms present at *HvFT1*, 89 cultivars, mainly European, and 20 inbred lines derived from Spanish landraces (Igartua et al., 1998). They were classified into 60 winter and 49 spring types, according to their genetic constitution at vernalization and photoperiod genes (Table S1). These genotypes constitute a representative sample of barley germplasm available to European breeders.

#### HvFT1 phenotypic effect

Several biparental populations used in past studies were reassessed to account for possible phenotypic effects of the polymorphisms at *HvFT1* (Table 1). In some cases, these effects were already described in the references cited. In others, further genotyping allowed a better resolution of the QTLs or the discovery of previously unknown polymorphism. QTL x QTL interaction analyses were done using the *unbalanced analysis of variance* option implemented in Genstat 14 (Payne et al., 2009), following a factorial model with the markers closest to the QTL peaks and “environment” as factors. The field experiments of the population Beka x Mogador are explained in the publication by Cuesta-Marcos et al. (2008).

**Table 1:** **Biparental populations analyzed in this study**.

**Population**	**Type**	**Number of lines**	***HvFT1* polymorphism**	**References**	**Present study**
Henni x Meltan	DH	118	Promoter	Borràs-Gelonch et al., 2010	Genotyping of *HvFT1* promoter
SBCC154 x Beatrix	DH	168	Intron and CNV	Unpublished	QTL analysis, genotyping of *HvFT1*
Beka x Mogador	DH	120	CNV	Cuesta-Marcos et al., 2008	New markers in *HvFT1* region and CNV. QTL interaction

#### Gene expression analysis

Seven spring cultivars and two landrace-derived inbred lines, selected to represent *HvFT1* CNV types, were used for gene expression analysis. Also, eight doubled haploid (DH) lines of the population Beka x Mogador (Table 2) were used for this purpose. Several major flowering time genes were segregating in this population (Cuesta-Marcos et al., 2008). To focus only on variation at *HvFT1*, DH lines were selected with spring (Beka) alleles at *VrnH1*, *VrnH2*, and *PpdH2.* Variation in *HvCEN*, the most important factor determining flowering time in this population, was also considered. We aimed at having two plants per *HvCEN*-*HvFT1* haplotype, but found only one for the Mogador-Mogador class (an extra plant was allocated to the Beka-Mogador class). *PpdH1* was not segregating in this cross. Both parents carry the recessive, long-day insensitive allele.

**Table 2:**
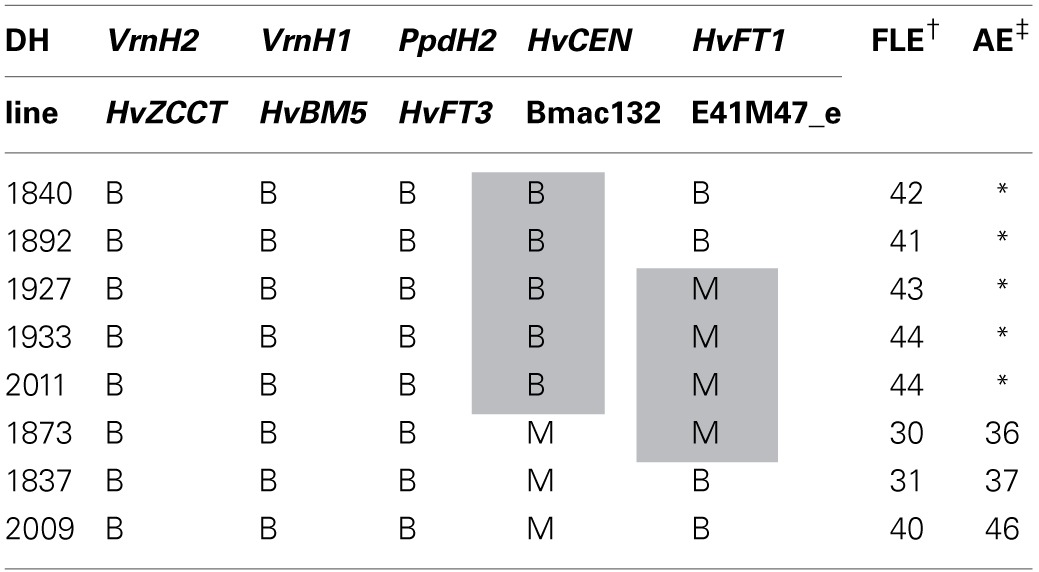
**Haplotypes for major flowering time genes and markers closest to QTLs (see Cuesta-Marcos et al., 2008) for selected doubled haploid (DH) lines of the population Beka × Mogador**.

Alleles conferring lateness are highlighted in gray. Dates for developmental stages in the pot experiment used for gene expression analysis are also included.

B, Beka; M, Mogador.

^†^FLE: days from sowing to 50% of plants with flag leaf expanded.

^‡^AE: days from sowing to 50% of plants with emerged awns.

^*^more than 48 days, as the experiment was terminated at that date and the plants had not reached awn emergence yet.

### Sequence polymorphisms at major genes

DNA sequence polymorphisms for the 109 accessions were screened with allele-specific primers of candidate genes. *VrnH1* was scored based on the size of the first intron of its candidate *HvBM5A* (Yan et al., 2003; von Zitzewitz et al., 2005). Alleles were classified according to Hemming et al. (2009); *VrnH2* was evaluated as presence of *HvZCCT-Ha* and *HvZCCT-Hb* (Karsai et al., 2005). *PpdH1* was genotyped using SNP22 in the *CCT* (*Constans, Constans-like, TOC1*) domain of its candidate gene *HvPRR7*, after digestion with *BstU I* (Turner et al., 2005). *PpdH2* was scored as presence of the *HvFT3* gene as reported by Casao et al. (2011). Regarding *VrnH3*, two indels in the promoter and two SNPs in the first intron of the *HvFT1* gene were assessed (Casas et al., 2011). *HvCEN*, candidate gene for *Mat-c* or *Eam6* was partially sequenced in 24 genotypes. The haplotypes are identified as reported by Comadran et al. (2012).

It was not possible to assess *HvFT1* polymorphism directly at the Beka x Mogador DH population (Cuesta-Marcos et al., 2008) because the sequences of the parental alleles were conserved and they differed only in copy number (2 Beka, 1 Mogador). The population was reanalyzed based on the new information found (parents polymorphic at *HvFT1* for CNV). Two microsatellite markers, in the *HvFT1* region, were mapped (EBmac0603 and AF022725A), to provide better resolution of the flowering time QTL found in this region.

### *HvFT1* CNV analysis by qPCR

Genomic DNA was isolated from frozen barley leaves using the NucleoSpin Plant II kit (Macherey-Nagel, Germany) and used as template for CNV analysis by qPCR in an ABI 7500, essentially as described by Nitcher et al. (2013) with some modifications. Briefly, 100 ng genomic DNA were mixed with 2 μM of each primer and 10 μl of 2X Power SYBR Green Supermix (Thermo Fisher Scientific, Waltham, MA). The PCR program comprised 10 min at 95°C, 40 cycles of 10 s 95°C and 50 s 60°C, and a melting curve stage. Number of copies of the first exon of *HvFT1* was tested in all 109 genotypes. Morex was selected as the calibrator genotype and *SNF2* as the housekeeping gene (Yan et al., 2002). Then, in a subset of lines, two other *HvFT1* regions (promoter and exon 3) and three other genes close to *HvFT1* (*UCW118*, *UCW123*, and *UCW120*) were tested as reported by Nitcher et al. (2013). Efficiency for each primer pair was obtained by serial dilutions of barley genomic DNA and it was taken into account for CNV calculation (Weaver et al., 2010). Efficiencies for *SNF2*, *HvFT1*-promoter, *HvFT1*-exon1, *HvFT1*-exon3, *UCW118*, *UCW120*, and *UCW123* were 0.95, 0.96, 0.92, 0.95, 0.96, 1.03, and 0.85, respectively.

### Growth conditions for expression studies

Barley plants used for expression analysis were grown in pots of 11.5 (diameter) × 16.0 (height) cm with a mix of peat, sand, and perlite. Six seeds of one genotype were sown per pot. After emergence, they were thinned to three seedlings per pot. The plants were grown in a growth chamber, under long-day conditions (16 h light, 250 μE m^−2^ s^−1^, 20°C, 60% relative humidity/8 h dark, 16°C, 65% relative humidity) for 7 weeks. There were three pots per genotype, which were used as replicates.

Two experiments were carried out with samplings at two different times. In the first experiment, leaf tissue was harvested in the middle of the light period, after 8 h light, as reported by Kikuchi et al. (2009). In the second experiment, to maximize circadian expression of *HvFT1* (Turner et al., 2005) harvesting took place 2 h before dark, after 14 h light. In both cases, leaf tissue (last expanded or flag leaf) was harvested and frozen immediately in liquid nitrogen before tissue homogenization (Mixer Mill model MM 400, Retsch, Germany). At each sampling time, three samples were analyzed per treatment and genotype. Each sample came from a different plant and pot.

### mRNA extraction, cDNA synthesis, and gene expression analysis

For qPCR analysis of *HvFT1* expression levels, 1 μg of total RNA (purified using the NucleoSpin RNA Plant kit, Macherey-Nagel) was transcribed to cDNA by using the SuperScript III reverse transcriptase and 2.5 μM poly(dT)_20_ primer according to the manufacturer's instructions (Invitrogen). The reaction mixture for qPCR and the PCR program have been previously described. cDNA was quantified using a Nanodrop system (Thermo Fisher Scientific) and equal amounts were used for all samples. *Actin* was selected as the housekeeping gene (Trevaskis et al., 2006), and expression of *HvFT1* and *HvCEN* was analyzed using the same primers as in Yan et al. (2006) and Comadran et al. (2012), respectively. Efficiencies for *Actin*, *HvFT1*, and *HvCEN*, were 0.97, 0.86, and 0.96, respectively.

## Results

### Survey of *HvFT1* CNV

**Figure 1:**
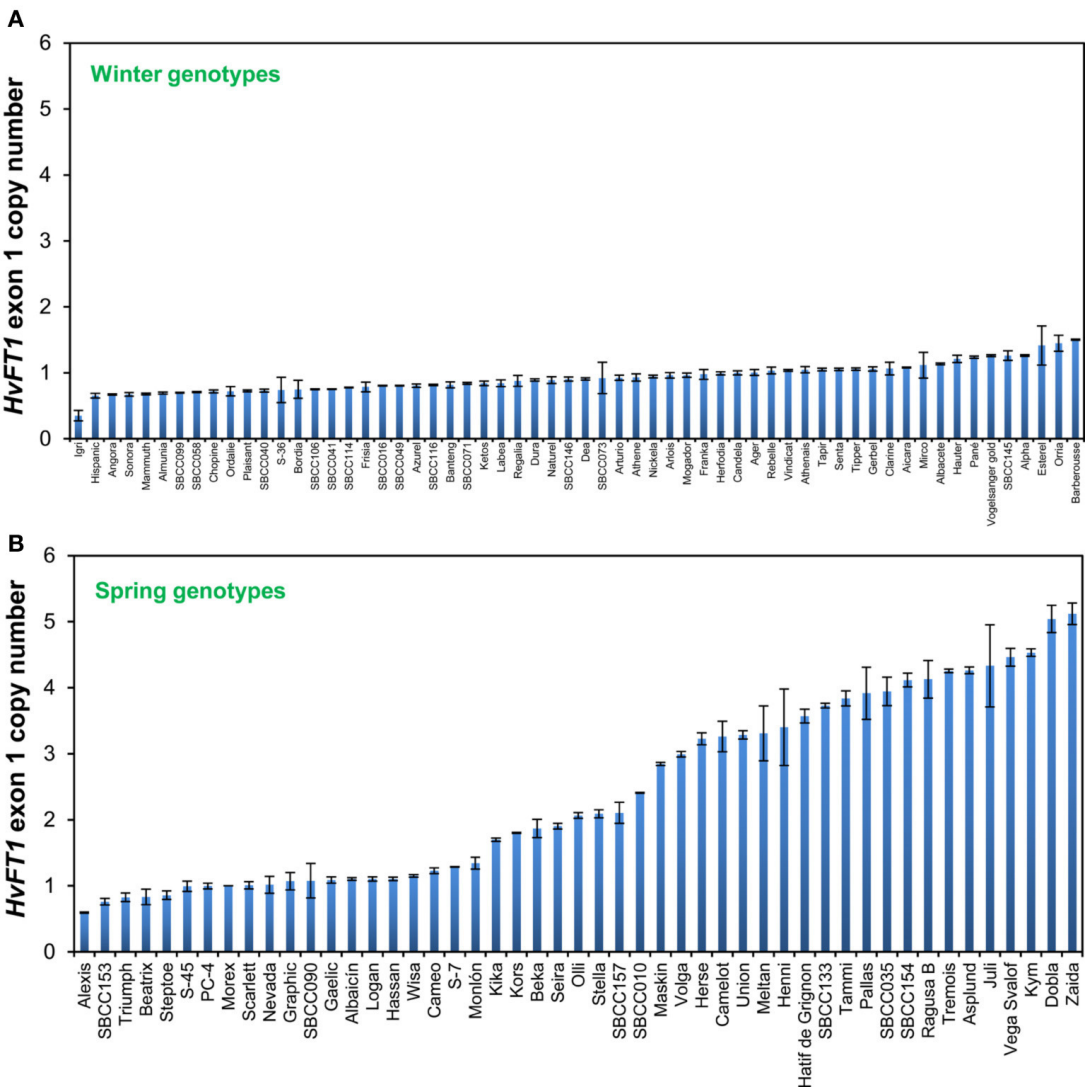
**Determination of copy number variation for the first exon of *HvFT1* in winter (A) and spring or facultative (B) barleys**. Bars represent means ± s.e.m. Morex was used as the calibrator genotype (copy number = 1). SBCC, Spanish Barley Core Collection.

Winter and spring genotypes were classified as follows: winter genotypes carry a functional *VrnH2* allele and a winter or intermediate allele in *VrnH1* (wild type *vrnH1*, *VrnH1-6*, or *VrnH1-4*). Spring or facultative lines have been classified as those with a spring allele in *VrnH1* or lines in which *VrnH2* is absent. With regard to *HvFT1*, there were accessions representing all possible combinations of intron and promoter polymorphisms, both within the winter and spring groups (Table 3). The combination of the “late” promoter with the “early” intron, however, was the most frequent. *HvFT1* exon 1 copy number ranged from 0.35 ± 0.13 in the winter cultivar Igri to 5.12 ± 0.28 in the spring cultivar Zaida (Figure 1). The results for several genotypes were intermediate between two classes, and were assigned to classes based on pedigree information when possible. Genotypes with several copies of *HvFT1* presented allele combinations typical of both winter and spring cultivars at all the *Vrn* and *Ppd* genes, with one exception, *VrnH2*. No genotype with the *VrnH2* gene present had more than one *HvFT1* copy.

**Table 3:** **Number of spring and winter accessions classified according to *HvFT1* haplotypes defined by polymorphisms at the promoter (indel 1- indel 2), first intron and number of copies of exon 1**.

**Promoter**	**Intron**	**Number of copies**	
		**1**	**>1**
**SPRING ACCESSIONS**			
135–146	AG	3	14
139–142	AG	1	1
135–146	TC	3	2
139–142	TC	14	11
**WINTER ACCESSIONS**			
135–146	AG	9	
139–142	AG	7	
135–146	TC	6	
139–142	TC	38	

It is remarkable that all of the winter barley cultivars, i.e., with a dominant *VrnH2* allele, had only one copy of the first exon. Barberousse, the last winter genotype in Figure 1, had 1.50 ± 0.01 copies, which we considered as a single copy. On the other hand, 28 out of the 49 spring cultivars analyzed contained more than 1.70 copies of exon 1 of *HvFT1*, which we have considered as multiple copies.

**Figure 2:**
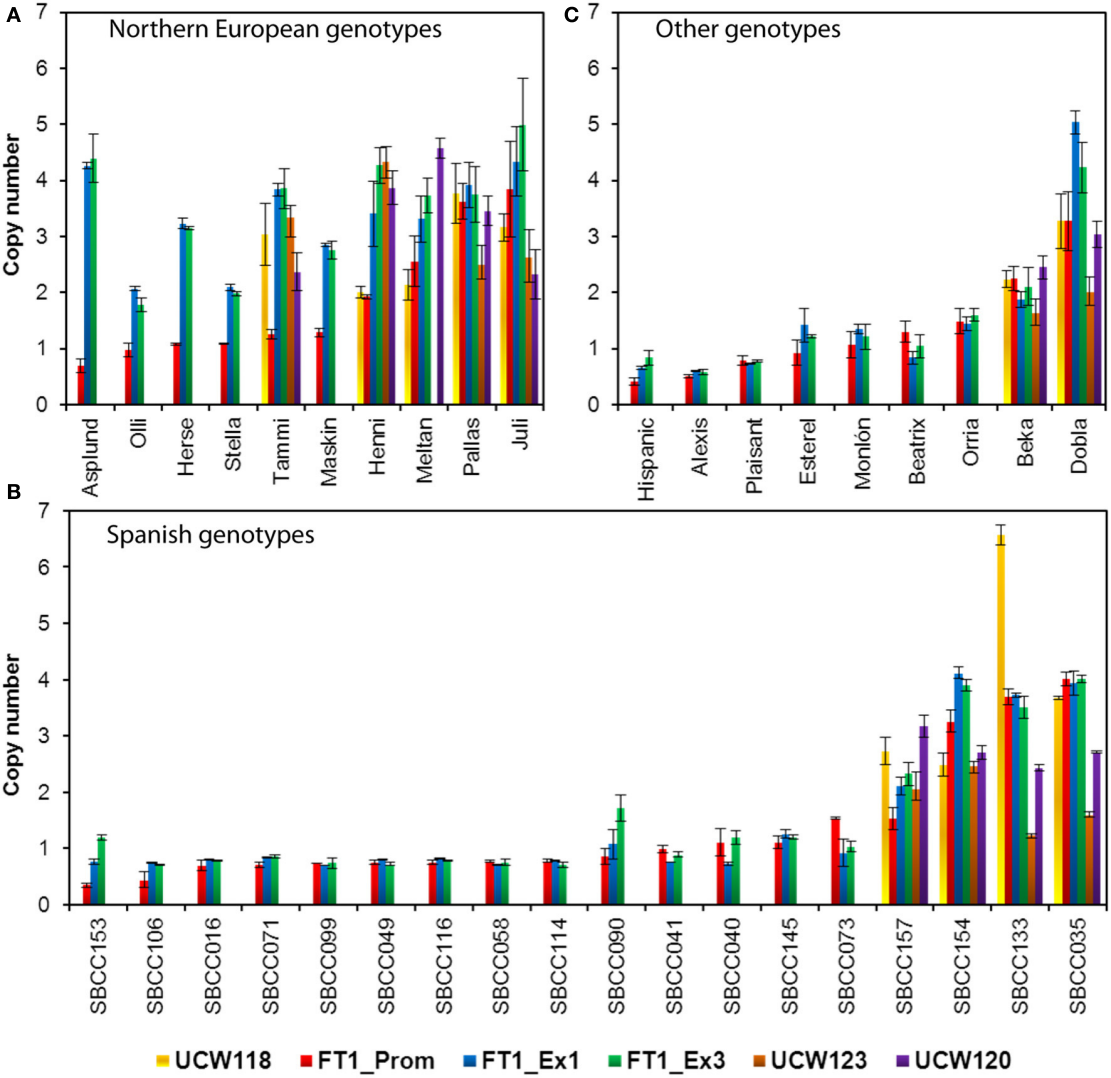
**Analysis of copy number variation for *HvFT1*(promoter, exon 1, and exon 3), and for regions upstream (*UCW118*) or downstream (*UCW123* and *UCW120*) of *HvFT1* on barley genotypes of Northern European (A), Spanish (B), and other origins (C)**. Bars represent means ± s.e.m. Morex was used as the calibrator genotype (copy number = 1). SBCC, Spanish Barley Core Collection.

The study was extended to analyze CNV in other areas within or near the *HvFT1* gene, as in Nitcher et al. (2013). Thus, as well as qPCR primers for *HvFT1* exon 1 (amplifying the region of +200 to +293 bp downstream from the ATG start codon), we used primers for *UCW118* (nearest known gene in the flanking Morex BAC 455J22 upstream from *HvFT1*), *HvFT1* promoter (−727 to −656 bp upstream from the ATG), *HvFT1* exon 3 (+778 to +902 bp downstream from the ATG), *UCW123* (+6.6 kbp downstream from *HvFT1*), and *UCW120* (nearest known gene in the flanking Morex BAC 761F04 downstream from *UCW123*). This analysis was carried out in 10 barley varieties from Northern Europe (Figure 2A), 18 Spanish landraces (Figure 2B), and in another nine varieties from diverse origins (Figure 2C). Although all genotypes were not analyzed for all the genes, several results merit further attention. Two main patterns of *HvFT1* promoter/exon 1/exon 3 copy number were found in Northern European barleys: Asplund, Olli, Herse, Stella, Tammi, and Maskin contained one single copy of the promoter and multiple copies of exon 1 and exon 3, whereas Henni, Meltan, Pallas, and Juli contained multiple copies of promoter, exon 1, and exon 3. These different gene structures may affect gene functionality, as we will discuss later. CNV in other genes around *HvFT1* was only analyzed in 11 accessions. *UCW118* (next to the promoter), and *UCW123* and *UCW120* (next to the exon 3) were similar to the corresponding CNV in the adjacent regions, with the exception of Tammi, which contained multiple copies of *UCW118* but only one promoter. We do not provide results for *UCW120* in Meltan since the primers gave no amplification. Regarding the Spanish lines, only 4 out of 18 genotypes analyzed (SBCC157, SBCC154, SBCC133, and SBCC035) contained multiple copies of the promoter, exon 1 and exon 3. For the other genotypes analyzed, only Beka and Dobla presented several gene copies in the *HvFT1* promoter, exon 1, and exon 3.

### Effect of *HvFT1* polymorphism in populations

We have reassessed several populations to illustrate the effect of sequence and CNV polymorphism at *HvFT1*.

**Figure 3:**
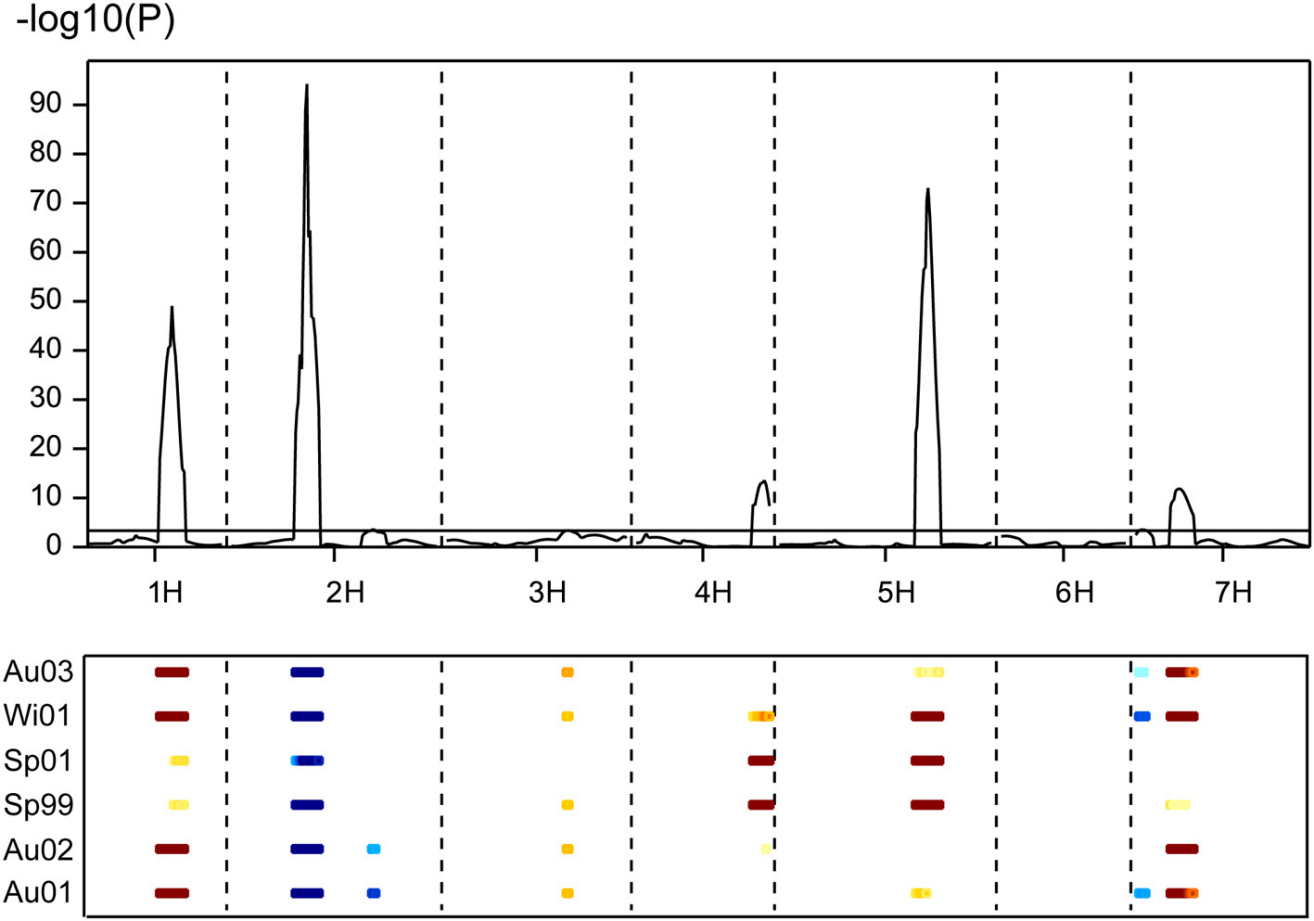
**Multi-environment QTL analysis for days to flowering from January 1st for six field experiments carried out with 120 doubled haploid lines and the parents of the population Beka x Mogador**. The peaks above the threshold (dashed line) indicate presence of QTL significantly affecting the trait. Data taken from Cuesta-Marcos et al. (2008), enriched with new markers on the 7HS chromosome. In the lower part of the figure, field trials are coded with Au (autumn sowing), Wi (winter sowing), or Sp (spring sowing) and two digits for the year; the colored dashes indicate the extent of the QTL and its direction: blue means that the early allele came from Mogador, yellow-brown from Beka, with intensity proportional to the size of the effect.

The population Beka x Mogador (Cuesta-Marcos et al., 2008) is a spring x winter population with very large flowering time variation. It has been reassessed because we have found that, although the sequence of *HvFT1* is conserved in both genotypes (1 distal mismatch in 2547 bp sequenced), it still presents CNV polymorphism: Beka has two copies of the gene, whereas Mogador has one. A small flowering time QTL in the vicinity of *HvFT1* was already reported, although the region was not well covered with markers in the original study. Close markers were now identified and genotyped in the population to increase coverage. The QTL already detected on 7HS in this cross was made more conspicuous with the markers introduced for this study (Figure 3), with the peak hinting at the *HvFT1* position, and a high significance [-log10(P) above 11]. In this case, Beka contributed the early allele, about 2.2 days earlier than the Mogador allele. If this QTL is truly due to the effect of *HvFT1*, it must be caused by differential effect of the number of copies. The possible effect of *HvFT1* CNV on the vernalization mechanism, as described by Nitcher et al. (2013), should have been evident in this population as an interaction of the QTL found at the *VrnH1*, *VrnH2* regions with the QTL at the *HvFT1* region, in trials without enough vernalization. This could have occurred in the three late sowings, in which the number of cooling degree days (a measure of vernalizing potential) was much lower than at the fall sowings (Table S1 in Cuesta-Marcos et al., 2008). The six field trials were reanalyzed for interactions between the five major QTL (*VrnH1*, *VrnH2*, *HvCEN*, *PpdH2*, *HvFT1*), using the closest marker to each peak, and dividing the six trials into fall sowings (November) and winter-spring sowings (late February to late March). The effect of *HvFT1* was clear across all trials, but did not interact much with other genes. There was just one possible interaction of *HvFT1* with *VrnH1* but in the fall-sown trials and not in the late sown trials. It was caused by a significant difference for the *HvFT1* alleles (with Beka, the one with two copies, inducing earliness) only in the presence of the winter (Mogador) allele at *VrnH1* (Table 4). The expected interaction between vernalization and *HvFT1* at the late-sown trials, however, was not detected at all. Therefore, we can conclude from this result that the possible promoting effect of the double *HvFT1* gene of parent Beka was not strong enough to affect the vernalization requirement of the winter lines of this population.

**Table 4:** **Probabilities of significance from three analyses of variance of flowering date recorded at six field trials, three fall-sown and three winter- or spring-sown in the Beka x Mogador population**.

**Source**	**All trials**	**Fall-sown**	**Winter- spring-sown**
	CPROB^*^		
Trial	**0.000**	**0.000**	**0.000**
*VrnH1*	**0.000**	0.280	**0.000**
*VrnH2*	**0.000**	**0.000**	**0.000**
*HvFT3*	**0.000**	**0.000**	**0.000**
*HvCEN*	**0.000**	**0.000**	**0.000**
*HvFT1*	**0.000**	**0.000**	**0.003**
Trial.*VrnH1*	**0.000**	0.237	**0.000**
Trial.*VrnH2*	**0.000**	0.511	**0.000**
Trial.*HvFT3*	0.092	**0.006**	0.632
Trial.*HvCEN*	**0.000**	0.612	**0.001**
Trial.*HvFT1*	0.766	0.248	0.698
*VrnH1.VrnH2*	**0.000**	0.424	**0.000**
*VrnH1.HvFT3*	0.489	0.527	0.644
*VrnH2.HvFT3*	0.947	0.934	0.811
*VrnH1.HvCEN*	**0.000**	**0.008**	**0.000**
*VrnH2.HvCEN*	0.786	0.072	0.074
*HvFT3.HvCEN*	0.817	0.305	0.276
*VrnH1.HvFT1*	0.097	**0.011**	0.699
*VrnH2.HvFT1*	0.564	0.760	0.652
*HvFT3.HvFT1*	0.330	0.509	0.477
*HvCEN.HvFT1*	0.109	0.078	0.548

The sources of variance are the trials plus five markers close to QTL peaks representing five major flowering time genes: VrnH1 (HvBM5), VrnH2 (HvZCCT), HvFT3 (Bmag382), HvCEN (Bmac132), and AFLP E41M47_e (HvFT1). Three way interactions are not shown, as none was significant. The analyses of variance were done on genotype means, taking as error the residual genotypic variance.

* Conditional probability of significance for each term, when added to a full model with the rest of terms already included. P-values below 0.05 highlighted in bold type.

**Figure 4:**
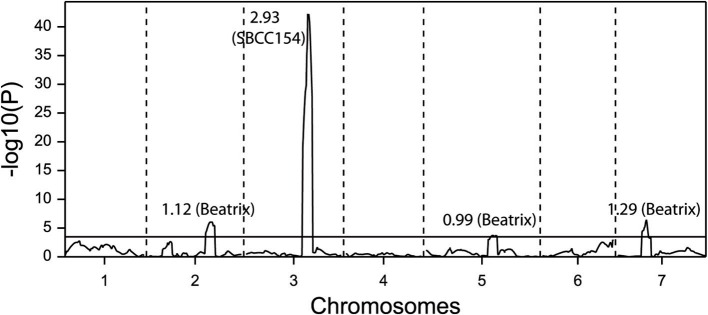
**QTL scan for flowering time at a field trial for a population of 168 doubled haploid lines from the cross SBCC154 x Beatrix**. The figures besides the QTL peaks indicate the size of the effect, with the early allele indicated in parentheses.

SBCC154 x Beatrix is a cross of two spring genotypes. The population was described by Hofmann et al. (2013), but the flowering time data have not been reported yet. SBCC154 has four copies of *HvFT1*, whereas Beatrix has only one. Both have the putatively late (139–142) promoter (Nitcher et al., 2013), whereas Beatrix has the early intron (TC) and SBCC154 the late one (AG). The *HvFT1* marker detected a significant QTL with an effect of 2.5 days, with Beatrix as the early allele (Figure 4).

The population Henni x Meltan (Borràs-Gelonch et al., 2010), a cross of two spring cultivars, was an example of polymorphism just at the promoter. Each genotype has four copies of *HvFT1* and, although they may have just two copies of the promoter; the parents present the same number of copies across the whole gene. The only polymorphism found was at the promoter, with Meltan carrying the early promoter (135–146) and Henni the late one (139–142). Confirming this expectation, the early allele of the QTL in Borràs-Gelonch et al. (2010) was contributed by Meltan, the difference being 23°C d (around 1–2 days).

### Expression analysis of *HvFT1* in selected spring barleys

**Figure 5:**
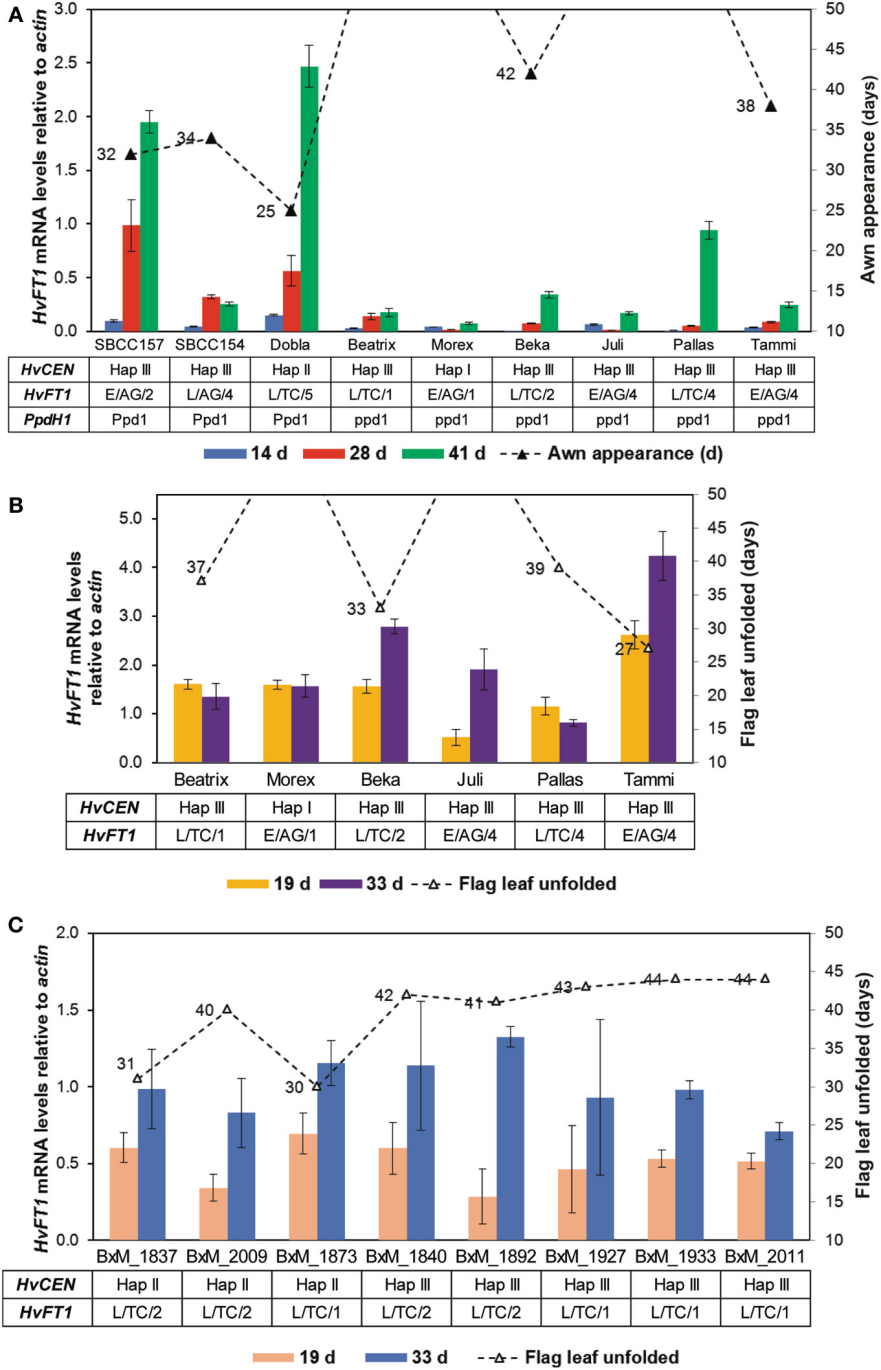
***HvFT1* expression analysis by qPCR**. In a first experiment **(A)**, leaf tissue was harvested in the middle of the light period (after 8 h light) 14, 28, or 41 days after sowing. Days until awn appearance (triangles) are shown for the whole duration of the experiment (50 days). *HvCEN*, *HvFT1*, and *PpdH1* haplotypes for each plant are also indicated. In the case of *HvFT1*, polymorphisms for promoter (E, “early”; L, “late”), intron 1 (AG or TC) and copy number variation (for exon 1) are shown. In the second experiment **(B,C)**, harvesting took place 2 h before dark (after 14 h light), 19 or 33 days after sowing. Days until full unfolding of the flag leaf (triangles) are shown for the whole duration of the experiment (50 days). As previously, *HvCEN* and *HvFT1* haplotypes for each plant are included. **(C)** Expression in selected doubled haploid lines of the Beka x Mogador population (BxM, see Table 2). Bars represent means ± s.e.m.

After finding CNV variation for *HvFT1*, the next step was to evaluate the effect of CNV variation on gene expression. If the effect of several copies of the gene was always as large as reported by Nitcher et al. (2013), then it should be detectable as a large increase of gene expression and a very early phenotype, overriding the effect of any other polymorphisms at *HvFT1*. This hypothesis was tested by evaluating gene expression on a set of spring and facultative cultivars representative of different *HvFT1* copy number alleles: Morex (reference genotype for one copy of *HvFT1*) and Beatrix as single *HvFT1* copy number, SBCC154, SBCC157, Dobla, Beka, Pallas, and Juli as examples of multiple promoter and exon 1 copies, and Tammi as representative of the genotypes with one promoter but multiple exon 1 copies (Table S1 and Figure 5A). *HvFT1* expression was found, in general, to increase according the developmental stage of the plants, although the expression levels differed widely among genotypes. At the second sampling date all genotypes, except Dobla, had not reached awn emergence yet. At this point, SBCC154, SBCC157, and Dobla, the earliest genotypes, displayed the highest *HvFT1* mRNA levels (awn emergence dates are included in Figure 5A). Apart from this observation, we did not observe much correlation between *HvFT1* expression and time to awn appearance. Interestingly, these three varieties had the dominant allele for *PpdH1*, whereas the rest had the recessive allele at this gene. Also, we could not find a clear correlation between number of *HvFT1* copies or sequence polymorphism and *HvFT1* mRNA expression. For example, Juli and Tammi contained multiple copies of *HvFT1* (four promoters and four genes in Juli, one promoter and four genes in Tammi), but both of them showed lower *HvFT1* expression than SBCC154, SBCC157, or Dobla, at the same sampling date. The influence of the difference in *HvFT1* promoter copies on *HvFT1* expression observed in Tammi with respect to Juli and Pallas will be discussed later. Thus, we can conclude that CNV has not a prevailing effect on *HvFT1* expression, over other types of polymorphisms at the same gene. It is not the main factor controlling *HvFT1* expression and flowering time (measured as awn appearance), and it depends largely on the genetic background, and polymorphisms at other genes, especially *PpdH1*, as has been reported previously in the literature in studies done before taking into account CNV (Turner et al., 2005; Hemming et al., 2008).

### Expression analysis of *HvFT1* in selected lines of the Beka x mogador population

In a second gene expression experiment, eight DH lines of the Beka x Mogador population were analyzed, together with some genotypes in common with the first experiment (Figures 5B,C). This population was found to contain a QTL for flowering time in *HvFT1* gene, as shown above. Individuals were selected according to CNV in *HvFT1*: Beka contained two copies while Mogador contained only one. qPCR analysis were performed as before, but this time the material was harvested 2 h before dark. Sampling time actually had a major effect on the detection of *HvFT1* expression: under these conditions, *HvFT1* mRNA levels were, in general, clearly higher than at the first experiment (Figure 5A), in which samples were harvested 8 h after the commencement of the light period, instead of 14 h. The varieties used in the first experiment, which had a recessive *ppdH1* allele, were also included for comparison. Apart from the clear induction of expression from a dominant *PpdH1* allele, we were not able to establish a clear relationship between *HvFT1* copy number, mRNA levels and flowering time for these plants (Figure 5B). *HvFT1* expression in the DHs was similar and stable during the experiment after 19 and 33 days. No significant differences were observed in *HvFT1* mRNA levels between the two alleles as main effects, although there was a significant interaction (*P* = 0.022) between the alleles at *HvCEN* and *HvFT1*. At the second sampling date, the plants with the Beka allele in *HvFT1* showed significantly higher expression than plants with Mogador allele, only if the allele at *HvCEN* also came from Beka. The Mogador allele at *HvCEN* had a major effect on earliness, and these lines (1837, 1873, and 2009) flowered markedly earlier than the others (flag leaf unfolding dates are included in Figure 5C).

Another interesting result of the second experiment was that Tammi showed the largest *HvFT1* expression after 33 days, and also flowered the earliest. It seems that Tammi has a more effective *HvFT1* gene to promote flowering than the others. This could be related to the fact that Tammi, as indicated above, presented four copies of the *HvFT1* gene but only one copy of the promoter, whereas Juli and Pallas, for example, have four copies of both promoter and exon 1.

### Expression analysis of *HvCEN* in the Beka x mogador population and in selected spring barley genotypes

**Figure 6:**
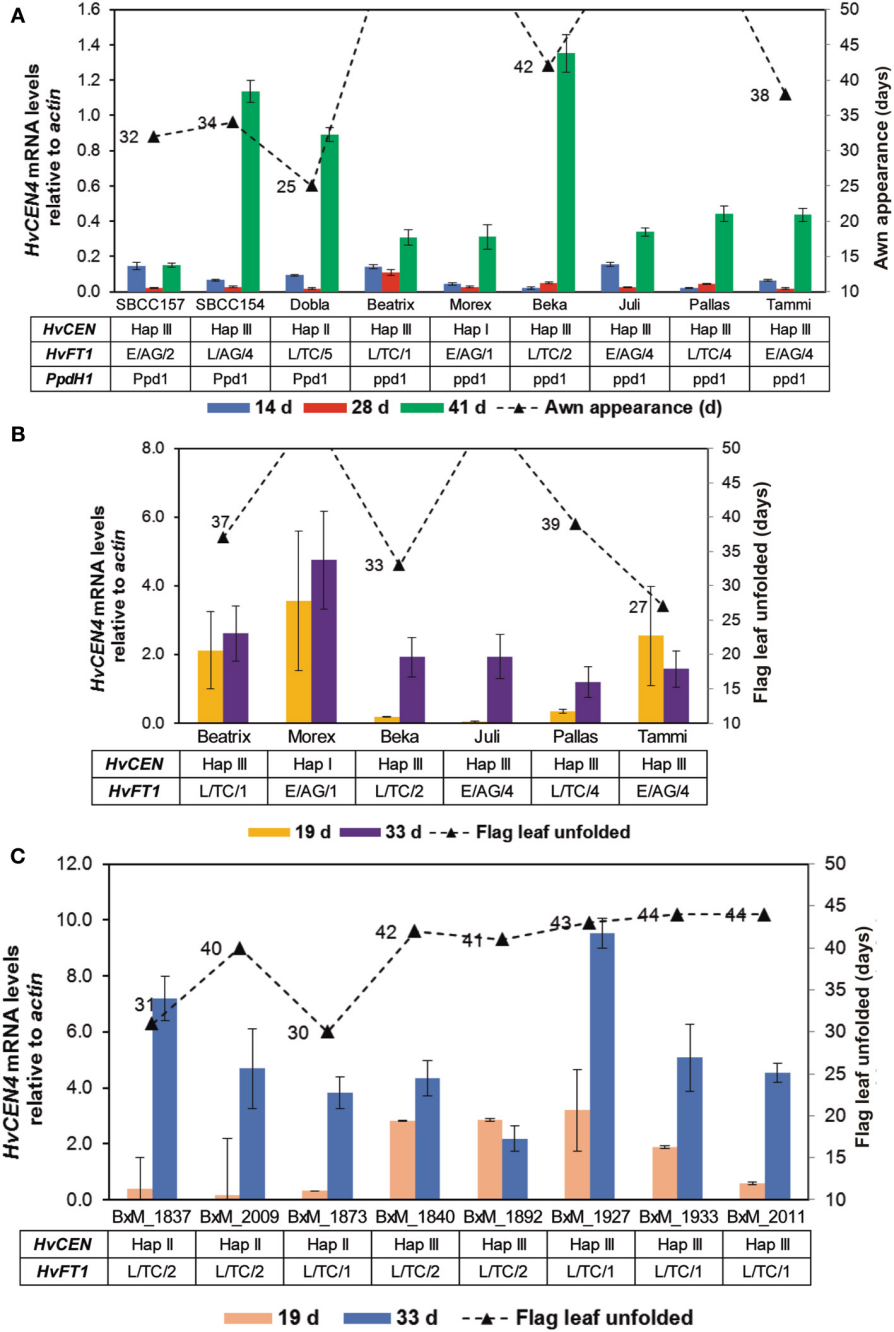
***HvCEN* expression analysis by qPCR**. *HvCEN* mRNA levels were quantified using the same conditions as for Figure 5. **(A)** leaf tissue was harvested in the middle of the light period (after 8 h light) 14, 28, or 41 days after sowing. **(B,C)**, harvesting took place after 14 h light, 19 or 33 days after sowing. **(C)** Expression in selected doubled haploid lines of the Beka x Mogador population (BxM, see Table 2). Bars represent means ± s.e.m.

As we have shown previously, CNV and different haplotypes for *HvFT1* are not sufficient to establish a clear relationship between them, *HvFT1* mRNA levels and awn appearance. For example, the presence of a dominant *PpdH1* allele was found to enhance *HvFT1* expression in different spring genotypes (Figure 5A). Additionally, CNV in *HvFT1* did not have a clear role to determine flag leaf unfolding in the Beka x Mogador DHs (Figure 5C). For these reasons, we decided to analyze the mRNA levels of another gene involved in the establishment of flowering time, *HvCEN*, a paralog of *FT* and *TFL1* in *Arabidopsis* (Kobayashi et al., 1999). We used the same cDNAs as for the previous expression studies (Figure 6). We included haplotype information for some genotypes about *HvCEN* in Table S1 for comparison. As observed for *HvFT1*, our results suggest that *HvCEN* mRNA levels are not the main responsible to explain awn appearance (Figure 6). *HvCEN* expression dramatically increased when samples were harvested 14 h after dawn instead of 8 h (Figures 6A–C), as we observed for *HvFT1*. Another remarkable observation is that *HvCEN* expression also changed during the development, in disagreement with previously observed results for *HvCEN* expression (Comadran et al., 2012). For example, Morex contained significantly higher *HvCEN* mRNA levels than Tammi, but Morex did not flower after 50 days and awns in Tammi appeared after 33 days.

## Discussion

### Distribution and phenotypic effect of *HvFT1* polymorphisms over barley germplasm

In this paper, we analyzed the extent of *HvFT1* CNV and its effect on *HvFT1* expression in more than 100 different spring and winter barley genotypes, mainly European, including some landraces from the Spanish Barley Core Collection (SBCC) (Igartua et al., 1998). Recently, Nitcher et al. (2013) demonstrated that the *FT1* allele present in the barley genetic stock BGS213, which carried several copies of *HvFT1*, showed earlier transcriptional up-regulation of *FT1*, and was associated with a dominant spring growth habit. Cultivar Tammi, from Finland, was the donor of the mutation present in that genetic stock (Franckowiak and Konishi, 1997). This allele is not frequent and was reportedly found only in spring cultivars originating from regions of extremely high latitude or high altitude (Takahashi and Yasuda, 1971). We confirmed the finding by Nitcher et al. (2013) that the Tammi/BGS213 allele was characterized by having a single copy of the promoter and several copies of the transcribed region. This kind of allele was present in a group of 6 six row Northern European cultivars, some of them related by pedigree (Baumer and Cais, 2000; Chiapparino et al., 2006). The origin of this allele could be traced back to primitive cultivar Asplund, which is in the parentage of Tammi and Herse, both showing alleles with multiple exon copies and one promoter (Figure 2). This allele seems to be particularly beneficial at high latitudes because both parents of Tammi (Asplund and Olli) come from the Northern limits of the barley cultivation range (Manninen and Nissila, 1997).

We found several copies of *HvFT1* in accessions from apparently disconnected germplasm groups, like Scandinavian and Spanish landraces. Although the sample examined is not enough to derive definitive conclusions, the distribution of CNV alleles over type of growth habit haplotypes is intriguing. Multiple copies at *HvFT1* occurs only in genotypes that do not have winter growth habit. This suggests a possible disadvantageous agronomic effect of the presence of several copies of the gene in interaction with *VrnH2*. A possible mechanism that provides a plausible explanation for this is that the increased copy number of *HvFT1* is epistatic to winter alleles for *VrnH1* and *VrnH2* (as reported by Nitcher et al., 2013). Muñoz-Amatriaín et al. (2013) have recently shown that high levels of CNVs are found in the barley genome, around 9.5% in coding genes, at increasing frequencies as distance to the centromere increases. This is a widespread phenomenon, contributing to phenotypic variation in barley. *HvFT1* CNV may be neutral in spring genotypes, but breeders could have selected against this duplication when breeding winter barley to avoid early transition to reproductive growth and exposure of reproductive tissues to damaging low temperatures. However, we have shown that this epistatic mechanism is not universal, as it does not occur in the Beka x Mogador population.

The reassessment of three biparental populations representing polymorphisms of different kind identified QTL in the region of *HvFT1*. Previous studies also shed some light on the possible phenotypic effect of *HvFT1*. The QTL found in the cross SBCC145 x Beatrix (Ponce-Molina et al., 2012), whose peak coincides with the gene, must be due to polymorphisms at the promoter region, as both parents have one copy of the gene and same first intron sequence. The early allele was contributed by SBCC145, a Spanish landrace from the Canary Islands, which carries the “early” promoter. It must be pointed out, however, that SBCC145 has a distinct promoter, with additional polymorphisms compared to others, similar to cultivars Dairokkaku (Casas et al., 2011) and Meltan. The cross SBCC154 x Beatrix offered the opportunity to contrast two different polymorphisms (promoter for SBCC145 x Beatrix, first intron, and CNV for SBCC154 x Beatrix) against a common parent (Beatrix). This population was tested at a single trial sown in autumn, in the same field as the population SBCC145 x Beatrix, and the result was the presence of a QTL, exactly at the marker used to genotype *HvFT1*, with SBCC154 as the late allele. In this case, high copy number (SBCC154) was late compared to one copy, but this could be due to an effect of SBCC154 carrying the “late” intron. Therefore, any acceleration of flowering that might be produced as a consequence of high copy number was secondary to the lateness associated to the sequence polymorphism. It must be noted that *PpdH1* was segregating in this population. However, flowering occurred too early to allow for a significant effect of *PpdH1*, which was not detected as QTL.

In the cross SBCC016 x Esterel (Casas et al., 2011), each genotype had one copy of *HvFT1*, Esterel has the “early” intron (TC) and SBCC016 the “late” one (AG). Accordingly, the early allele of the QTL, which also peaked at the gene itself, was contributed by Esterel. Hayes et al. (1993) and Borràs-Gelonch et al. (2012) reported a QTL in this region for the population Steptoe x Morex. Although the size of the effect was not large, in both studies the Morex allele was significantly later. Both parents have just one copy of the gene, and a mixture of late and early polymorphisms at the promoter and the intron. It seems that the intron effect is stronger, as Steptoe carries the early (TC) allele at this position.

A QTL in the region of *HvFT1* was also detected in the classical studies carried out in the Igri x Triumph population (Laurie et al., 1995), with the Triumph allele conferring earlier flowering, although the nucleotide sequences were identical (Yan et al., 2006). CNV has been recently identified in this population, with two copies of the *HvFT1* gene in Triumph (R. Nitcher, personal communication). This result would match our findings in the Beka x Mogador population. Nevertheless, we could not reproduce this result, since the Triumph seed we analyzed had only one copy of the gene. We cannot discard that the samples analyzed are different, because this cultivar is known under two different names, Trumpf in Germany and Triumph in the UK, where it was reselected from somewhat heterogeneous seed (van Harten, 1998).

In summary, these findings reported in the literature, combined with the results presented in this study, reveal that detection of flowering time QTL in the region of *HvFT1* in biparental populations representing all kinds of polymorphisms at *HvFT1* (promoter, first intron, and CNV) is common. There is no functional proof that this gene is responsible for all the QTL, but it is a good candidate. An alternative explanation could be the presence, at least in some cases, of an additional flowering time gene closely linked to *HvFT1*. In any case, we have to wait until there are either functional proofs or increasing evidence from other populations to declare that *HvFT1* is the responsible for the variation observed. We expect that the catalog of polymorphisms presented in this study will help other researchers to contribute information to clarify the issue.

The survey of *HvFT1* polymorphisms allows us to conclude that *HvFT1* (*VrnH3*) is far from being effectively fixed in cultivated barley, as stated up to now (Stracke et al., 2009; Comadran et al., 2012). This statement is probably true if one only considers the allele responsible for the huge phenotypic effect observed by Nitcher et al. (2013) on a winter barley, which confirmed observations of the seminal work by Takahashi and Yasuda (1971). The effects that we have found in different populations (this study), association studies (Casas et al., 2011) and also reported in the literature (previous paragraphs) point at smaller phenotypic effects that are linked to all types of polymorphisms in this gene (promoter, first intron, CNV). These effects, however, cannot be easily extrapolated to different genetic backgrounds.

### CNV and gene expression

The commencement of the reproductive stage in barley and the duration of the time period until flowering are controlled by a variety of factors that act interactively. In temperate cereals like wheat and barley, flowering is promoted by long days. In barley, the up-regulation of *VRN3/FT1* appears to be the main trigger for the initiation of flowering (Faure et al., 2007), although an alternative view is that its role may be to accelerate inflorescence development and reduce the time taken from double-ridge to head emergence (Sasani et al., 2009). Its expression is tightly regulated, repressed by *VRN2* under long days (Hemming et al., 2008), which actually competes with *CO* to bind to protein complexes that activate *FT* (Li et al., 2011). Also in response to long days, *FT1* expression is promoted by CO-like proteins (Li et al., 2011; Campoli et al., 2012) and the pseudo response regulator *PpdH1* (Turner et al., 2005; Faure et al., 2007). This promotion may occur by interaction of CO with the promoter of *FT*, as happens in *Arabidopsis* (Tiwari et al., 2010; Li et al., 2011; Andrés and Coupland, 2012).

The findings of this study add to mounting evidence pointing at a complex control of the timing of head emergence that cannot be easily reduced to a simple scheme. On one hand, *HvFT1* displays a variety of polymorphisms in regions that are compatible with a regulatory role. On the other hand, its expression pattern is compounded by its nodal position, downstream of the vernalization and long day pathways, whose genes also have large influence on duration of plant development.

The first gene expression experiment results confirmed the induction of *HvFT1* expression by the long day pathway, irrespective of the polymorphisms present at *HvFT1*. The genotypes that reached first awn appearance in that experiment were all those having a sensitive/dominant *PpdH1* allele, particularly at the second sampling date. This date is probably the most meaningful because all *PpdH1* genotypes reached awn appearance just a few days later. This result agrees with other reports where *HvFT1* expression in *ppdH1* background was markedly lower (Turner et al., 2005; Hemming et al., 2008; Kikuchi et al., 2009). Therefore, we can conclude that gene duplication of *HvFT1* does not always ensure higher expression and, in any case, the scale of its effect is minor compared to the induction by *PpdH1*. The case of high *HvFT1* expression in presence of *ppdH1* described in Nitcher et al. (2013) seems an exception, probably due to a unique genetic background.

Our results confirm that the triggering of events at the meristem leading to flowering is not determined just by *HvFT1* expression. There must be additional factors that probably need the presence of *HvFT1* product to interact with. We cannot rule out, however, that copy number is related to a dosage effect, precisely by the different genetic background in each genotype, which may lead to differences in the induction of *HvFT1*. A beneficial increase in dosage is actually one of the evolutionary forces explaining the conservation of gene duplications, particularly for genes that mediate the interaction between the organism and the environment, as reported for *Ppd-B1* and *Vrn-A1* in wheat by Díaz et al. (2012), or for genes with dosage-sensitive functions, owing to protein-protein interaction (Innan and Kondrashov, 2010). *HvFT1* corresponds to the first class, and may as well be part of the second. CNV is widespread in plants (Zmieńko et al., 2014), and certainly in barley (Muñoz-Amatriaín et al., 2013), but its effects vary in each case. For instance, in barley, the powdery mildew resistance allele *mlo-11* acts by disrupting its expression through accumulation of non-functional copies of the gene upstream of the wild-type copy (Piffanelli et al., 2004). On the other hand, the effect of the freezing tolerance locus *FrH2* in barley depends on the number of *CBF* genes transcripts produced, that are proportional to the number of copies present in the gene cluster identified as responsible for this QTL (Pasquariello et al., 2014). The plant immunity locus *GER4*, also in barley, is also a cluster of tandemly duplicated genes. In this case, the enhanced transcript dosage was proposed as the evolutionary driving force for the local expansion and functional redundancy of the locus (Himmelbach et al., 2010).

The results of Nitcher et al. (2013) and the earliness induced by the double copy “Beka” allele in the Beka x Mogador population point in the direction of a dosage effect. However, the direct comparison of *HvFT1* expression figures for different alleles is hampered by the large influence of the alleles at *PpdH1* on *HvFT1* and at *HvCEN* on flowering time. The analysis of the eight sister lines of the Beka x Mogador population indicates a slightly earlier flowering and higher *HvFT1* expression of lines with the Beka allele only in presence of the Beka allele at *HvCEN* (spring, Hap III), but the evidence is too small to declare that we have found a clear dosage effect.

These results led us to speculate that the most efficient version of the barley gene could be one with a single conserved promoter, and variable number of transcribed regions, as seen in cultivar Tammi, or one promoter and one copy of the gene. Other versions of the gene, with several promoters and transcribed regions may take longer to be induced. Indeed, we do not know whether the multiple copies of *HvFT1* are functional or not. They seem to be expressed in Tammi/BGS213, but there is no evidence for other alleles. If they are, the presence of several full and functional copies of the gene may affect differently the expression of the gene, depending on the concentration of the promoting signal. If it is low, its dilution over several copies may delay transcription, whereas an abundant promoting signal would enhance transcription proportionally in alleles with several copies. The special case of Tammi/BGS213 indicates either that the transcription of several copies is triggered by a single promoter, or that the additional copies of the gene are placed under the control of other promoters and thus escape the regulatory control of the wild-type gene. For other multiple copy alleles, with equal number of promoters and transcribed regions, we can speculate that the copies are full and perfect replicates of the original gene, and that the expression of them all is affected by the same processes.

The difference in overall gene expression between the two experiments agrees with the reports on pattern of diurnal oscillation of *FT1* expression during the day (Turner et al., 2005; Kikuchi et al., 2009). Actually, the same observation can be made for *HvCEN* (Figure 6). This information, combined with the sequence analogy between the two genes, suggests also that *HvCEN* could be subject to circadian rhythm.

### Interactions of *HvFT1*

The DH lines (Figures 5C, 6C) presented very different dates to reach a developmental stage (flag leaf unfolding in this case) in the presence of rather similar levels of *HvFT1* expression. As pointed out before, in this case the cause for differences in development seems to lie on the allele present at *HvCEN*. It is clear that accelerated development in the presence of the Mogador *HvCEN* allele (Hap II) did not depend on higher expression of *HvFT1*. Therefore, early development in these lines must be caused by some mechanism acting in parallel to *HvFT1*, either through protein-protein interaction with *HvFT1* or by a combined effect with *HvFT1* by focusing on the same targets.

Actually, the interaction between *HvFT1* and *HvCEN* is by no means unexpected. *HvCEN* and *HvFT1* are probably paralogs as suggested by their high sequence identity (59%), and indeed have been annotated as members of closely related protein families before (Higgins et al., 2010; Andrés and Coupland, 2012; Comadran et al., 2012). Therefore, it is likely that their products display similar interactive patterns with other proteins. There are many evidences in the literature pointing at the interaction of FT proteins with other proteins in the flowering promotion pathway. Ahn et al. (2006) reported that the family of small proteins coded by *FT* and homologous genes act as “either scaffolds or regulators of signaling complexes” in *Arabidopsis*. The bZIP transcription factor FD also seems to play a central role in flowering (Wigge et al., 2005). More recently, Jaeger et al. (2013) put forward a model to explain flowering control in *Arabidopsis* in which TFL1 (the product of another paralog gene) competed with FT to form the complex with the FD gene product, needed to trigger floral meristem specification. Some evidence for this kind of process in cereals was found by Li and Dubcovsky (2008), who detected interaction between the proteins of *TaFT* (the *FT* homolog of bread wheat) and *FD-like2*, and also in rice (Taoka et al., 2011), although in this case the interaction needed the intermediation of a third protein. In barley, however, *FD* orthologs can only be predicted in terms of sequence similarity.

A search in the Protein Data Bank revealed that, besides the FT-FD interaction, *Arabidopsis* homodimers of FT and also TFL1 have been reported. Thus, it is also conceivable that proteins coming from genes as similar as *HvFT1* and *HvCEN* may form heterodimers as well, making the speculation about the interaction between the products of these two genes more plausible. The *HvCEN* polymorphism, which translates to a non-synonymous mutation (Pro135Ala), is located in a solvent-exposed protein loop (Comadran et al., 2012). However, with the structural evidence at hand, this loop does not directly participate in any dimeric interface and therefore nothing can be concluded about its molecular role in the interface.

The role of *HvFT1* may actually go beyond the duration of developmental phases. There is recent evidence about dramatic agronomic effects of the ortholog of *FT* in tomato, *SFT* in interaction with *SP* (*SELF PRUNING*, itself an ortholog of *HvCEN* and *HvTFL1*). *SFT* is in a “dose-dependent epistatic interaction” with *SP* which results in a modification of plant architecture that can be optimized to produce higher yields (Jiang et al., 2013). This dose-dependent action of *SFT* also opens the ground for speculation about possible dose-dependent action of multiple copies of *HvFT1* and its agronomic outcome.

## Conclusions and further work

The main conclusions of this study are:

- A wide survey of barley germplasm revealed that *HvFT1* duplication was only observed in spring and facultative barleys that do not possess a functional *VrnH2* allele.
- Two models of *HvFT1* duplication were observed, one that includes the promoter and the gene, the other only the transcribed region. Higher gene expression seems associated only to the second one.
- There are flowering time QTL on the region of *HvFT1* in different populations, representing all types of polymorphism at *HvFT1*, promoter, first intron and CNV.
- Analysis of *HvFT1* expression and phenotypic effects showed that they depend on gene polymorphisms but also on genetic background.

Plant breeders must be able to fully harness the development of cereal plants to be able to respond to the challenges of climate change. In this study, we present the case to state that *HvFT1* has been a hot spot to fine tune barley adaptation to environmental conditions, and will have to be given due consideration by breeders to create future cultivars.

The role of other genes in triggering flowering initiation, possibly in interaction with *HvFT1*, seems a very rich area which deserves more research. In particular, further avenues to attempt to resolve the precise sequence or structural variation in *HvFT1* which causes early or late flowering,- particularly for the Tammi allele will require crossing different *HvFT1* alleles into a common genetic background, maintaining fixed *PpdH1* and *HvCEN*. This could be combined with BAC sequencing for the entire *HvFT1* region, for key alleles.

## Author contributions

Ernesto Igartua, Ana M. Casas and M. P. Gracia conceived the experiments. Jorge Loscos performed qPCR analysis. Ana M. Casas and Jorge Loscos characterized the flowering time genes. Ernesto Igartua and Jorge Loscos performed computational analysis. Bruno Contreras-Moreira performed the bioinformatics searches and helped with the interpretation of the data. All authors wrote and approved the manuscript.

### Conflict of interest statement

The authors declare that the research was conducted in the absence of any commercial or financial relationships that could be construed as a potential conflict of interest.

## Supplementary material

Table S1 **Barley accessions surveyed in this study classified according to the genetic constitution at vernalization and photoperiod genes**. Countries of origin and row number are also indicated.
